# Entry points of nutrient arteries at risk during osteotomy of the lesser metatarsals: a fresh cadaveric study

**DOI:** 10.1186/s13047-018-0288-8

**Published:** 2018-08-08

**Authors:** Ichiro Tonogai, Fumio Hayashi, Yoshihiro Tsuruo, Koichi Sairyo

**Affiliations:** 1 Department of Orthopedics, Institute of Biomedical Science, Tokushima University Graduate School, 3-18-15 Kuramoto, Tokushima, 770-8503 Japan; 2 Department of Anatomy and Cell Biology, Institute of Biomedical Science, Tokushima University Graduate School, 3-18-15 Kuramoto, Tokushima, 770-8503 Japan

**Keywords:** Lesser metatarsal, Nutrient artery, Osteotomy, Cadaver

## Abstract

**Background:**

Osteotomies of the lesser (second to fourth) metatarsals are often used to correct forefoot deformities. However, certain areas of the lesser metatarsals where arteries may be prone to damage during surgery, and the resulting nonunion and delayed union could cause serious problems. This study sought to identify the nutrient arteries of the lesser metatarsals and to determine how osteotomy could injure these vessels.

**Methods:**

Enhanced computed tomography scans of 21 ft (male, *n* = 10; female, *n* = 11; mean age 78.6 years at the time of death) were assessed. Twenty-one lower limbs in 21 cadaveric specimens were injected with barium via the external iliac artery, and the points at which the nutrient arteries entered the lesser metatarsals were identified on axial and coronal images.

**Results:**

Each nutrient artery entered the lateral or medial plantar aspect of the lesser metatarsal in the middle third (just proximal to the middle point of the metatarsal) or proximal third obliquely from a distal direction. The mean ± standard deviation (SD) distances from the dorsal plane of the second, third, and fourth metatarsals to the point of entry of the nutrient artery in the axial plane were 8.2 ± 1.5, 7.6 ± 1.2, and 7.6 ± 1.5 mm, respectively. The mean ± SD distances from the distal epiphysis to the point of entry of the nutrient artery into the second, third, and fourth metatarsals in the coronal plane were 3.3 ± 1.1, 3.1 ± 1.0, and 2.8 ± 1.2 mm, respectively. The mean ± SD distances from the distal epiphysis to the point of entry of the nutrient artery into the second, third, and fourth metatarsals in the coronal plane were 46.0 ± 5.2, 40.9 ± 2.6, and 39.1 ± 3.7 mm, respectively. The mean ± SD distances from the proximal epiphysis to the entry point of the nutrient artery into the second, third, and fourth metatarsals in the coronal plane were 23.8 ± 4.7, 25.8 ± 4.3, and 25.0 ± 3.2 mm, respectively.

**Conclusions:**

Distal metatarsal osteotomies might be safer than shaft or proximal osteotomy to avoid disruption of the nutrient artery, leading to delayed consolidation of the osteotomy and nonunion.

## Background

Osteotomies of the lesser metatarsals are used to treat a variety of pathologies of the foot, including metatarsalgia and metatarsophalangeal subluxation or dislocation [1]. Many osteotomy techniques have been described to shorten or elevate the lesser metatarsals, including oblique shaft osteotomy [2, 3], segmental midshaft osteotomy [4, 5], and proximal metatarsal osteotomy [6–9].

Complications following osteotomy of the lesser metatarsals, which include nonunion and delayed union [10], have been attributed to iatrogenic disruption of the blood supply [11, 12]. DeSandis et al. reported a nonunion rate of 6.6% after a mean 12.9 months of follow-up after shaft osteotomy of a lesser metatarsal [1], while Galluch et al. reported that the nonunion rate after lesser metatarsal shaft osteotomy was 0.8% [5]. Spence et al. reported that only 24% of patients achieved bony union and the remaining 76% were considered to have nonunion after proximal osteotomy of a lesser metatarsal [4], while Lee et al. reported 2 cases of delayed union and 1 of nonunion in 85 ft (in 65 consecutive patients) that underwent proximal osteotomy of a lesser metatarsal [13].

Nonunion and delayed union of a lesser metatarsal may be related to disruption of the blood supply and has prompted recommendations to restrict the use of osteotomy. We have previously reported the point at which the nutrient artery enters the fifth lesser metatarsal [14], and the points at which the nutrient arteries enter the second, third, and fourth metatarsals have been discussed in a few reports [11, 12, 15]. However, there have been few studies with detailed description of the entry point of the nutrient artery into the lesser metatarsal.

The aims of this fresh cadaveric study were to identify the points at which nutrient arteries enter the second, third, and fourth metatarsals on axial and coronal enhanced computed tomography (CT) scans and to identify measures that could help to prevent injury to these arteries during osteotomy of the lesser metatarsals.

## Methods

This study was approved by the research board at our institution and included 21 ft of 21 fresh cadavers (male, *n* = 10; female, *n* = 11; mean age 78.6 years [range 48–100] at the time of death). Cadavers with a history or signs of previous ankle trauma or surgery, congenital or developmental deformity, or inflammatory arthritis were excluded.

The vessels were flushed with warm normal saline solution through a plastic catheter placed in the external iliac artery. Barium sulfate suspension (Barytester®, Fushimi Pharmaceutical Co., Inc., Marugame, Japan) was then injected into the external iliac artery with application of firm manual pressure as described in our previous reports [16–18]. Enhanced multi-slice computed tomography (CT) images (Somatom Emotion 16, Siemens Healthcare, Erlangen, Germany) of the lower extremities were obtained in 1.0-mm-thick axial slices. Coronal and axial images were reviewed at bone window setting (window, 2200; level, 200).

**Fig. 1:**
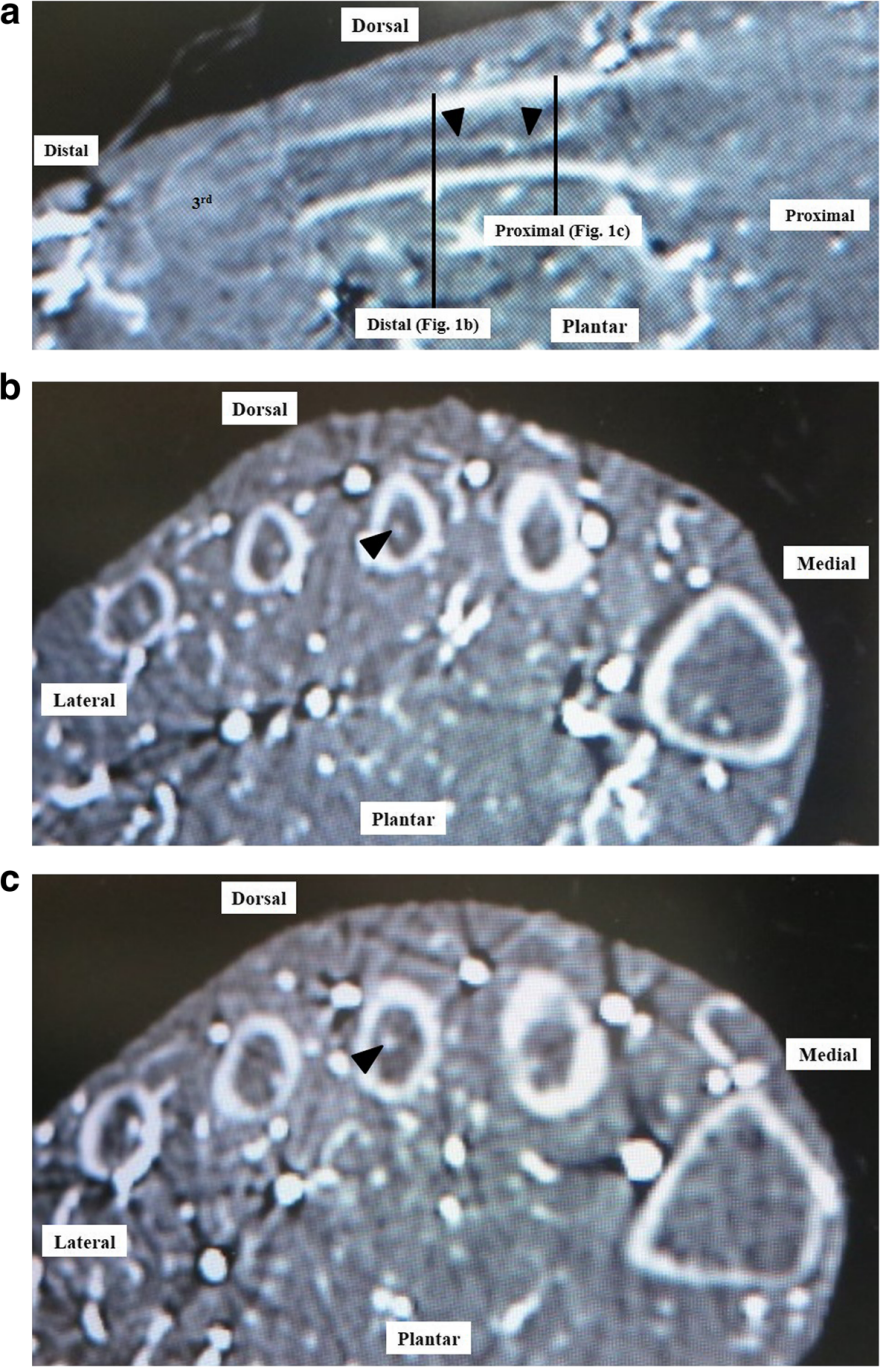
Enhanced CT images showing the nutrient artery course after entering the lesser metatarsal. The nutrient artery runs within the medullary canal distally and proximally in the sagittal plane (**a**) and distally (**b**) and proximally (**c**) in the axial plane. The arrow head indicates the nutrient artery

**Fig. 2:**
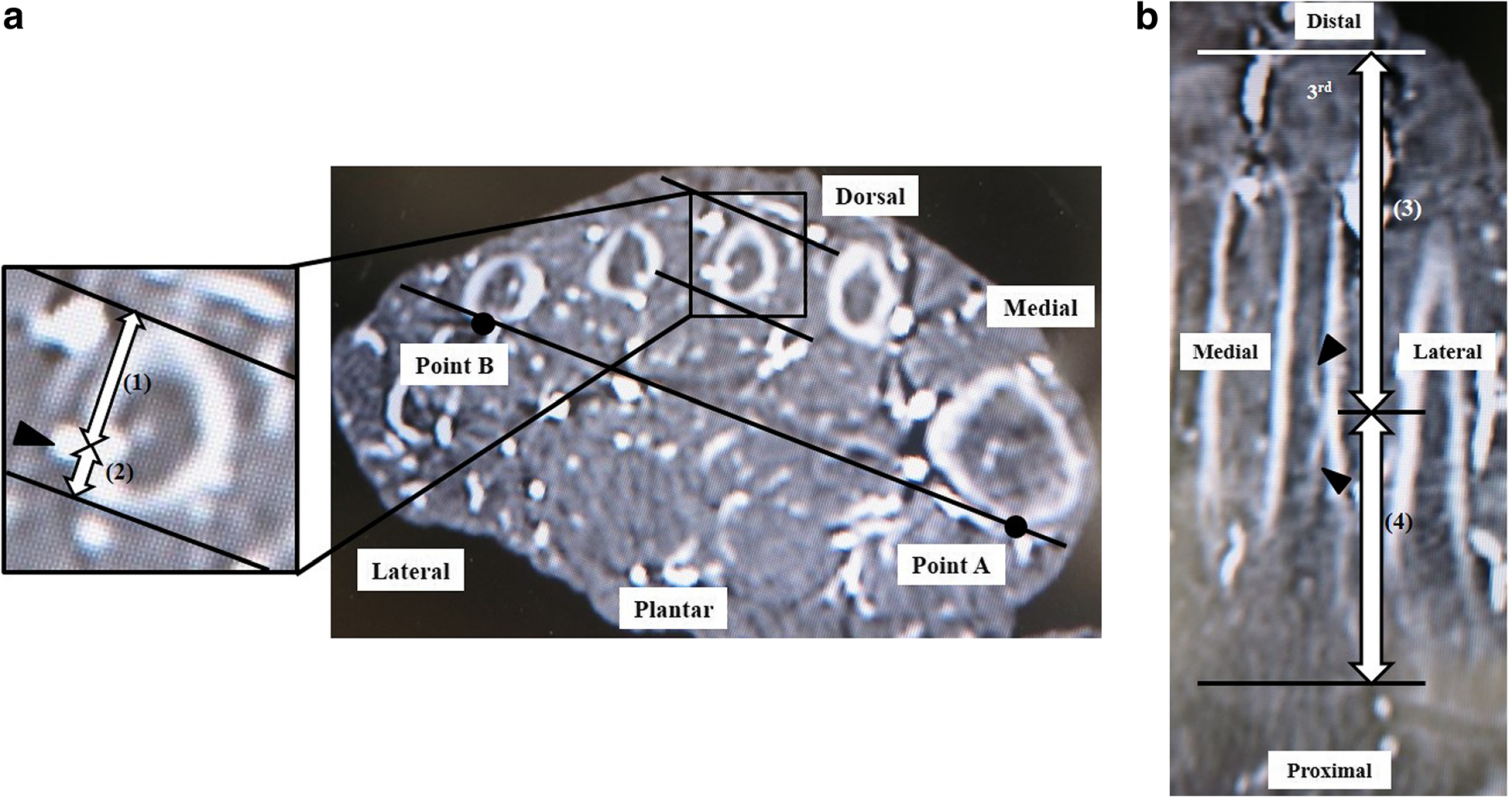
Enhanced CT images showing nutrient artery parameters in relation to the lesser metatarsals. **a** Distance from the dorsal plane parallel to a line between the most plantar point of the first metatarsal (point A) and fifth metatarsal (point B) to the point of entry of the nutrient artery into the lesser metatarsal (1) and the distance from a line between the most plantar point of the first metatarsal (point A) and fifth metatarsal (point B) to the point of entry of the nutrient artery into the lesser metatarsal (2) in the axial plane. **b** Distance from the distal epiphysis to the point of entry of the nutrient artery into the lesser metatarsal (3), the distance from the proximal epiphysis to the point of entry of the nutrient artery into the lesser metatarsal (4) in the coronal plane. The arrow head indicates the nutrient artery

**Fig. 3:**
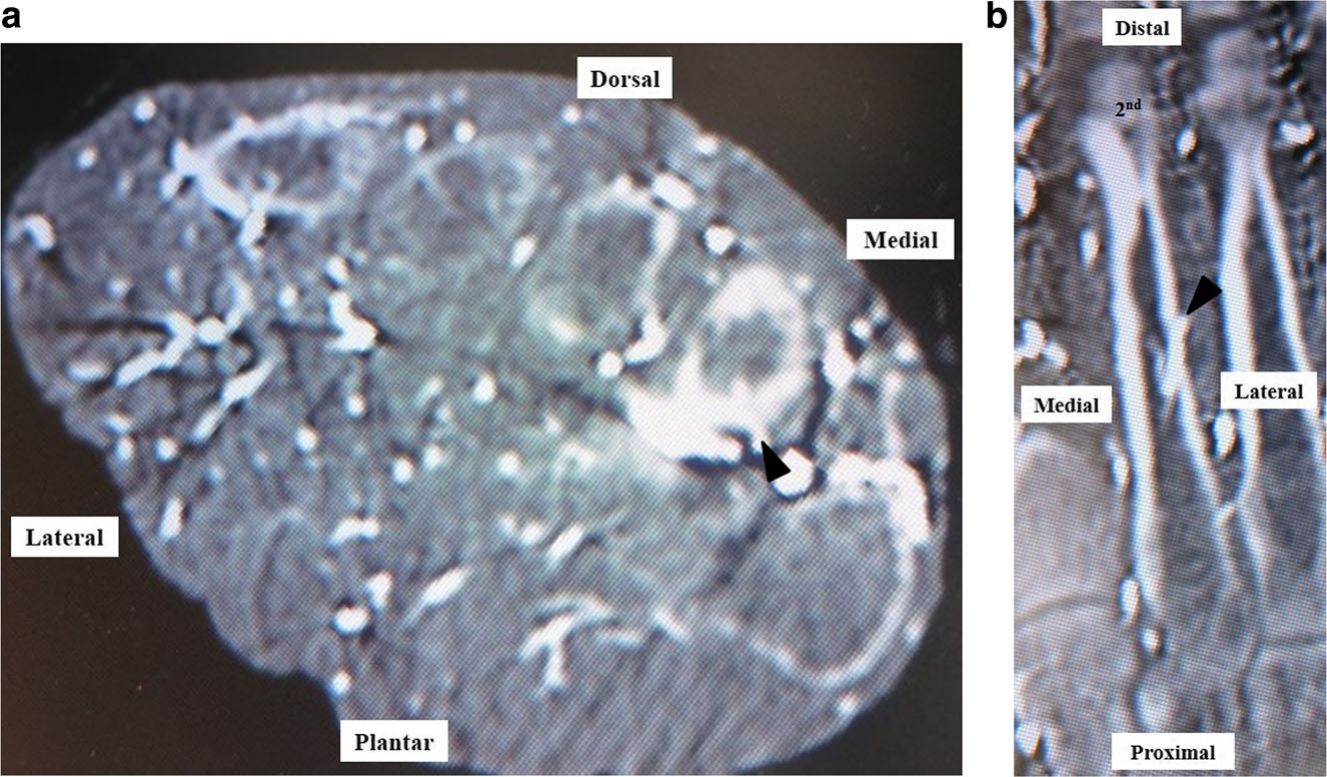
Enhanced CT images showing the nutrient artery entering the lesser metatarsal medially (**a**) In the axial plane and, (**b**) in the coronal plane. The arrow head indicates the nutrient artery

We confirmed the continuity of each nutrient artery after it entered the second, third, or fourth metatarsal. The nutrient artery divides into distal and proximal branches [12, 15], so we also confirmed that it ran distally and proximally within the medullary canal (Fig. 1a–c). As outlined in Table 1, the following parameters were measured: (1) the distance from the dorsal plane parallel to a line between the most plantar point of the first metatarsal (point A) and fifth metatarsal (point B) to the point of entry of the nutrient artery into the lesser metatarsal in the axial plane (Fig. 2a); (2) the distance from a line between the most plantar point of the first metatarsal (point A) and fifth metatarsal (point B) to the point of entry of the nutrient artery into the lesser metatarsal in the axial plane (Fig. 2a); (3) the distance from the distal epiphysis to the point of entry of the nutrient artery into the lesser metatarsal in the coronal plane (Fig. 2b); and (4) the distance from the proximal epiphysis to the point of entry of the nutrient artery in the coronal plane (Fig. 2b). Measurements were made in triplicate by two independent orthopedic surgeons (F.H. and K.S) who were blinded to the purpose of the study, and the first author (I.T.) did not results to measurement. Mean values were calculated. Data are expressed as mean ± standard deviation (SD).

**Table 1:** Measurement parameters of nutrient artery entry points of the lesser metatarsals on CT

CT axial plane	(1) Distances from the dorsal tangential plane to the points of entry of the nutrient arteries into the second, third, and fourth metatarsals
	(2) Distances from the plantar tangential plane to the points of entry of the nutrient arteries into the second, third, and fourth metatarsals
CT coronal plane	(3) Distances from the distal epiphysis to the points of entry of the nutrient arteries into the second, third and fourth metatarsals
	(4) Distances from the proximal epiphysis to the points of entry of the nutrient arteries into the second, third and fourth metatarsals

*CT* computed tomography

## Results

A summary of the results is shown in Tables 2, 3 and 4. The mean ± standard deviation (SD) distances from the dorsal plane of the second, third, and fourth metatarsals to the points of entry of the nutrient artery in the axial plane (1) were 8.2 ± 1.5 mm (range 6.1–11.6), 7.6 ± 1.2 mm (range 6.0–11.0), and 7.6 ± 1.5 mm (range 5.5–10.6), respectively. The mean ± (SD) distances from the plantar plane of the second, third, and fourth metatarsals to the points of entry of the nutrient artery in the axial plane (2) were 3.3 ± 1.1 mm (range 1.0–5.0), 3.1 ± 1.0 mm (range 1.4–5.2), and 2.8 ± 1.2 mm (range 1.0–6.2), respectively. All nutrient arteries entered the lesser metatarsals from the plantar aspect, but some entered via the lateral aspect and others via the medial aspect (Fig. 3a, b). The nutrient arteries supplying the second, third, and fourth metatarsals entered from the lateral plantar direction in 15/21 (71.4%), 14/21 (66.7%), and 7/21 (33.3%) specimens, respectively.

**Table 2:** Measurements obtained for the nutrient artery of the second metatarsal in each specimen

Specimen	Sex, age (years), side of individual at death	Axial plane			Coronal plane		
		Distance (mm) from the point of entry of the second nutrient artery		Direction of the nutrient artery into the second metatarsal	Distance (mm) from the point of entry of the second nutrient artery		Entry point of the nutrient artery into the second metatarsal
		(1) Dorsal plane	(2) Plantar plane		(3) Distal epiphysis	(4) Proximal epiphysis	
1	Male, 70, Right	7.9	3.0	Plantar-lateral	46	24	Middle third
2	Male, 70, Right	6.7	4.8	Plantar-lateral	36	35	Middle third
3	Male, 77, Right	9.1	3.0	Plantar-lateral	43	26	Middle third
4	Female, 95, Left	10.5	1.7	Plantar-lateral	48	15	Proximal third
5	Female, 48, Left	9.1	3.0	Plantar-medial	52	29	Middle third
6	Female, 100, Left	9.8	2.7	Plantar-lateral	54	14	Proximal third
7	Male, 78, Right	8.9	3.6	Plantar-medial	47	19	Proximal third
8	Female, 70, Left	6.5	4.9	Plantar-lateral	44	23	Middle third
9	Male, 96, Right	11.6	1.0	Plantar-lateral	61	18	Proximal third
10	Male, 92, Left	7.1	4.5	Plantar-lateral	43	26	Middle third
11	Female, 69, Right	6.1	4.6	Plantar-lateral	40	31	Middle third
12	Female, 87, Right	7.3	1.7	Plantar-lateral	42	22	Middle third
13	Female, 78, Left	9.2	1.6	Plantar-medial	45	22	Proximal third
14	Female, 56, Left	8.5	4.1	Plantar-medial	51	25	Proximal third
15	Female, 80, Right	7.5	3.0	Plantar-lateral	44	24	Middle third
16	Female, 74, Left	6.1	4.3	Plantar-lateral	42	23	Middle third
17	Male, 73, Right	8.6	3.8	Plantar-medial	47	24	Middle third
18	Male, 93, Left	7.3	4.3	Plantar-lateral	43	26	Middle third
19	Male, 87, Right	10.5	2.3	Plantar-medial	47	23	Proximal third
20	Male, 77, Left	8.1	3.4	Plantar-lateral	43	27	Middle third
21	Male, 81, Right	6.4	5.0	Plantar-lateral	49	24	Proximal third
Mean ± SD	78 ± 12	8.2 ± 1.5	3.3 ± 1.1		46.0 ± 5.2	23.8 ± 4.7	

*SD* standard deviation

**Table 3:** Measurements for the nutrient artery that entered the third metatarsal in each specimen

Specimen	Sex, age (years), side of individual at death	Axial plane			Coronal plane		
		Distance (mm) from the point of entry of the third nutrient artery		Direction of the nutrient artery into the third metatarsal	Distance (mm) from the point of entry of the third nutrient artery		Entry point of the nutrient artery into the third metatarsal
		(1) Dorsal plane	(2) Plantar plane		(3) Distal epiphysis	(4) Proximal epiphysis	
1	Male, 70, Right	7.2	4.1	Plantar-lateral	41	26	Middle third
2	Male, 70, Right	11.0	1.3	Plantar-medial	44	25	Middle third
3	Male, 77, Right	8.1	3.2	Plantar-lateral	40	24	Middle third
4	Female, 95, Left	9.4	1.4	Plantar-lateral	39	23	Middle third
5	Female, 48, Left	7.2	3.3	Plantar-lateral	42	28	Middle third
6	Female, 100, Left	7.7	2.7	Plantar-medial	42	25	Middle third
7	Male, 78, Right	9.1	2.2	Plantar-medial	46	18	Proximal third
8	Female, 70, Left	7.4	3.8	Plantar-lateral	42	24	Middle third
9	Male, 96, Right	6.6	5.2	Plantar-lateral	42	34	Middle third
10	Male, 92, Left	8.3	4.1	Plantar-lateral	42	25	Middle third
11	Female, 69, Right	6.3	3.1	Plantar-lateral	35	34	Middle third
12	Female, 87, Right	6.8	3.6	Plantar-medial	44	16	Proximal third
13	Female, 78, Left	7.9	2.3	Plantar-medial	41	20	Proximal third
14	Female, 56, Left	8.3	2.6	Plantar-lateral	41	31	Middle third
15	Female, 80, Right	6.0	3.7	Plantar-lateral	36	28	Middle third
16	Female, 74, Left	5.3	4.2	Plantar-medial	38	26	Middle third
17	Male, 73, Right	7.2	3.6	Plantar-lateral	44	25	Middle third
18	Male, 93, Left	6.8	4.3	Plantar-lateral	39	28	Middle third
19	Male, 87, Right	6.7	4.1	Plantar-lateral	39	28	Middle third
20	Male, 77, Left	7.7	2.5	Plantar-medial	39	27	Middle third
21	Male, 81, Right	8.6	1.8	Plantar-lateral	44	28	Middle third
Mean ± SD	78 ± 12	7.6 ± 1.2	3.1 ± 1.0		40.9 ± 2.6	25.8 ± 4.3	

*SD* standard deviation

**Table 4:** Measurements for the nutrient artery that entered the fourth metatarsal in each specimen

Specimen	Sex, age (years), side of individual at death	Axial plane			Coronal plane		
		Distance (mm) from entry point of nutrient artery to		Direction of nutrient artery into fourth metatarsal	Distance (mm) from entry point of the fourth nutrient artery to		Entry point of nutrient artery into fourth metatarsal
		(1) Dorsal plane	(2) Plantar plane		(3) Distal epiphysis	(4) Proximal epiphysis	
1	Male, 70, Right	9.5	2.2	Plantar-medial	38	26	Middle third
2	Male, 70, Right	10.1	1.2	Plantar-medial	36	26	Middle third
3	Male, 77, Right	10.0	1.0	Plantar-medial	39	23	Middle third
4	Female, 95, Left	8.7	1.3	Plantar-medial	33	28	Middle third
5	Female, 48, Left	7.2	3.0	Plantar-lateral	39	28	Middle third
6	Female, 100, Left	6.5	3.0	Plantar-medial	38	28	Middle third
7	Male, 78, Right	6.0	4.1	Plantar-medial	39	23	Middle third
8	Female, 70, Left	5.5	4.9	Plantar-lateral	43	20	Proximal third
9	Male, 96, Right	10.6	1.0	Plantar-medial	48	27	Middle third
10	Male, 92, Left	9.2	3.3	Plantar-medial	41	24	Middle third
11	Female, 69, Right	6.4	6.2	Plantar-medial	39	29	Middle third
12	Female, 87, Right	8.1	3.5	Plantar-medial	40	19	Proximal third
13	Female, 78, Left	6.7	1.7	Plantar-lateral	35	25	Middle third
14	Female, 56, Left	6.9	3.5	Plantar-lateral	33	32	Middle third
15	Female, 80, Right	6.0	3.1	Plantar-lateral	38	24	Middle third
16	Female, 74, Left	6.2	2.8	Plantar-medial	42	20	Proximal third
17	Male, 73, Right	9.1	2.7	Plantar-lateral	44	21	Proximal third
18	Male, 93, Left	7.3	2.8	Plantar-medial	39	26	Middle third
19	Male, 87, Right	8.0	2.4	Plantar-medial	38	26	Middle third
20	Male, 77, Left	5.7	2.8	Plantar-medial	35	27	Middle third
21	Male, 81, Right	7.1	2.9	Plantar-lateral	46	24	Middle third
Mean ± SD	78 ± 12	7.6 ± 1.5	2.8 ± 1.2		39.1 ± 3.7	25.0 ± 3.2	

*SD* standard deviation

**Fig. 4:**
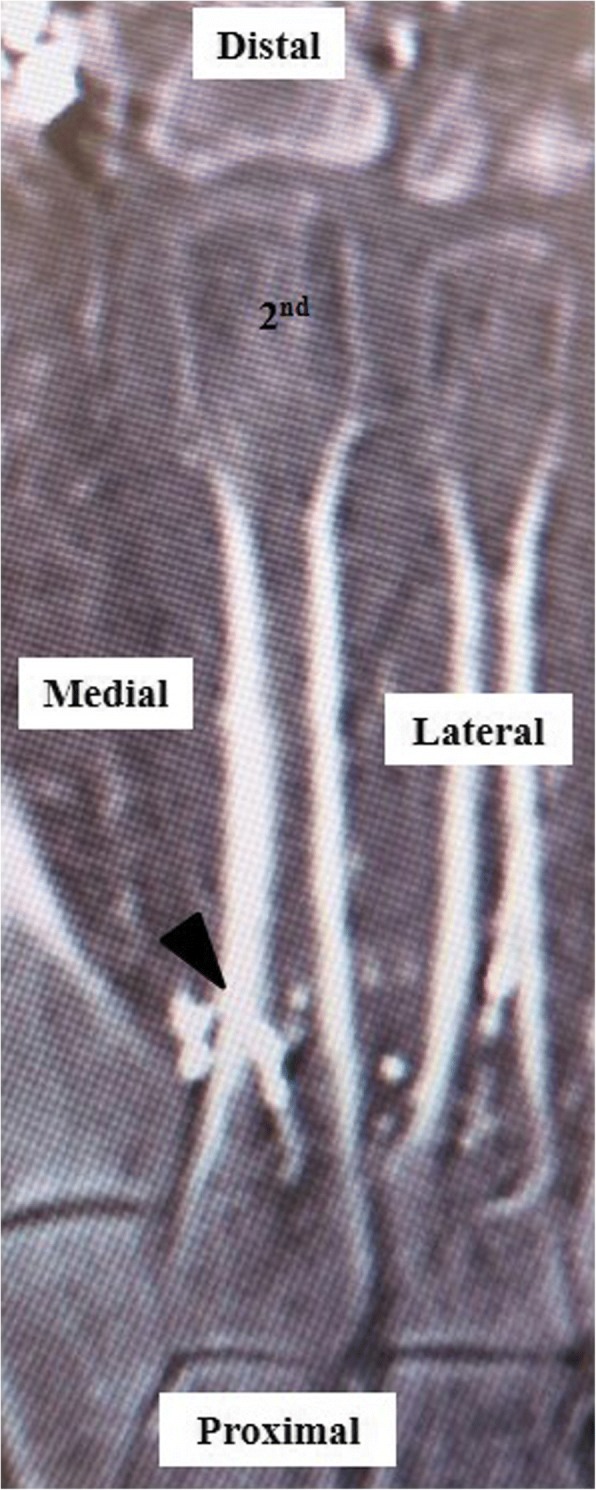
Enhanced CT showing the nutrient artery entering the lesser metatarsal near the mid-point. The nutrient artery enters the lesser metatarsal near the mid-point from a distal direction laterally and obliquely in the coronal plane. The arrow head indicates the nutrient artery

All nutrient arteries entered from the distal direction obliquely in the coronal plane (Fig. 4). The mean ± (SD) distances from the distal epiphysis to the points of entry of the nutrient artery into the second, third, and fourth metatarsals in the coronal plane (3) were 46.0 ± 5.2 mm (range 36–61), 40.9 ± 2.6 mm (range 35–46), and 39.1 ± 3.7 mm (range 33–48), respectively. The mean ± (SD) distances from the proximal epiphysis to the entry points of the nutrient artery into the second, third, and fourth metatarsals in the coronal plane (4) were 23.8 ± 4.7 mm (range 15–35), 25.8 ± 4.3 mm (range 16–34), and 25.0 ± 3.2 mm (range 19–32). Most of the nutrient arteries entered the second, third, and fourth metatarsals between the middle third and the proximal third, but some entered very near to the midpoint of the metatarsal (specimen 2 in the second metatarsal, specimen 11 in the third metatarsal, and specimen 14 in the fourth metatarsal; Fig. 4). The nutrient arteries entered the second, third, and fourth metatarsals at the level of the middle third in 13/21 (61.9%), 18/21 (85.7%), and 17/21 (81.0%) of the specimens, respectively. When the nutrient artery entered the second, third, or fourth metatarsal at the level of the middle third, the entry point was just proximal to the middle point of the metatarsal.

## Discussion

**Fig. 5:**
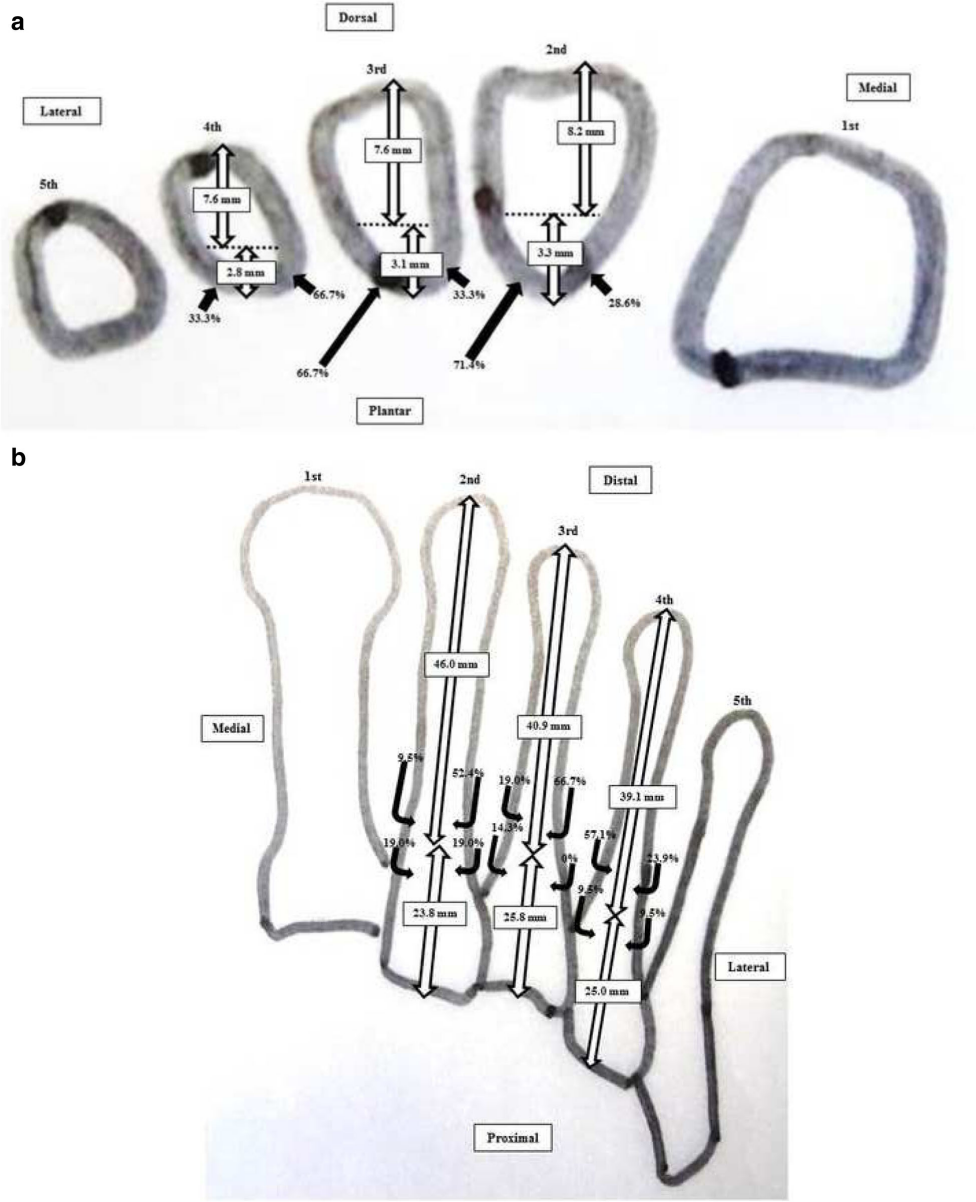
Diagrammatic a representations of the entry points of the lesser metatarsals in the axial (**a**) and coronal (**b**) pla

This study shows that the nutrient arteries do not enter into the second, third, or fourth metatarsal from a designated direction, that is, lateral or medial, and that the nutrient arteries supplying the second, third, and fourth metatarsals enter at the middle third (just proximal to the midpoint of the metatarsal) or proximal third distally from a medial or lateral plantar direction, as diagrammatic representions show (Fig. 5a, b). Our findings might imply that care is needed on both sides when performing an osteotomy involving a lesser metatarsal, and that the widely practiced distal metatarsal osteotomies, such as Weil and Schwartz, should be considered than shaft or proximal osteotomy to avoid disruption of the nutrient artery, leading to delayed consolidation of the osteotomy and nonunion.

Petersen et al. [12] and Sarrafian [15] have reported that a nutrient artery enters the second, third, or fourth metatarsal by penetrating the lateral diaphysis. In the present study, although many nutrient arteries entered the lesser metatarsals via the lateral aspect (second metatarsal, 71.4%; third metatarsal, 66.7%; fourth metatarsal, 33.3%), many also entered via the medial aspect. To our knowledge, this is the first fresh cadaveric study to show on enhanced CT that the entry point of the nutrient artery into a lesser metatarsal is sometimes from the medial direction.

Petersen et al. [11] and Rath et al. [12] reported that the nutrient artery transverses the cortex of the metatarsal in a distal direction in the coronal plane. In this study, the nutrient artery entered the lesser metatarsal obliquely in a distal direction in all cases. Moreover, Sarrafian reported that the pattern of entry of the nutrient artery into the second, third, and fourth metatarsals was similar [15], and our findings were consistent with that report.

Sarrafian also reported that the nutrient artery penetrates the lateral surface near the base in the coronal plane [15]. However, Petersen et al. reported that the nutrient artery enters the bone in the middle of the diaphysis (midshaft) [11]. In our study, some nutrient arteries entered the lesser metatarsal at the level of the middle third (just proximal to midpoint of the metatarsal) and others at the level of the proximal third. This variation might reflect ethnic differences in foot and ankle anatomy.

Our study had several limitations, in particular the small number of specimens used, which is inevitable because of the limited availability of fresh-frozen cadavers in Japan. Another limitation is that the intraosseous blood supply provided by the periosteal plexus and metaphyseal and epiphyseal vessels via anastomosis was not assessed, given that these arteries also supply blood to the lesser metatarsals. No intraosseous arteries smaller than the nutrient arteries were identified in this study.

## Conclusions

In conclusion, this study has shown that the nutrient arteries supplying the second, third, and fourth metatarsals enter at the middle third (just proximal to the midpoint of the metatarsal) or proximal third distally from a medial or lateral plantar direction. The point at which the nutrient artery enters a lesser metatarsal is important when performing osteotomy. Although shaft and proximal osteotomies are useful, they may interrupt blood flow in the nutrient artery and cause delayed union or nonunion. The widely practiced distal metatarsal osteotomies might be safaer than shaft or proximal osteotomy.

## Abbreviations

CT: Computed tomography
SD: Standard deviation
